# Long non-coding RNAs harboring miRNA seed regions are enriched in prostate cancer exosomes

**DOI:** 10.1038/srep24922

**Published:** 2016-04-22

**Authors:** Alireza Ahadi, Samuel Brennan, Paul J. Kennedy, Gyorgy Hutvagner, Nham Tran

**Affiliations:** 1 Centre for Human Centred Technology Design, University of Technology, Sydney; 2 Centre for Health Technologies, Faculty of Engineering and Information Technology, University of Technology, Sydney; 3 School of Life Sciences, Faculty of Science, University of Technology, Sydney; 4 Centre for Quantum Computation and Intelligent Systems, University of Technology, Sydney; 5 The Sydney Head and Neck Cancer Institute, Sydney Cancer Centre, Royal Prince Alfred Hospital, Australia

## Abstract

Long non-coding RNAs (lncRNAs) form the largest transcript class in the human transcriptome. These lncRNA are expressed not only in the cells, but they are also present in the cell-derived extracellular vesicles such as exosomes. The function of these lncRNAs in cancer biology is not entirely clear, but they appear to be modulators of gene expression. In this study, we characterize the expression of lncRNAs in several prostate cancer exosomes and their parental cell lines. We show that certain lncRNAs are enriched in cancer exosomes with the overall expression signatures varying across cell lines. These exosomal lncRNAs are themselves enriched for miRNA seeds with a preference for *let-7* family members as well as miR-17, miR-18a, miR-20a, miR-93 and miR-106b. The enrichment of miRNA seed regions in exosomal lncRNAs is matched with a concomitant high expression of the same miRNA. In addition, the exosomal lncRNAs also showed an over representation of RNA binding protein binding motifs. The two most common motifs belonged to ELAVL1 and RBMX. Given the enrichment of miRNA and RBP sites on exosomal lncRNAs, their interplay may suggest a possible function in prostate cancer carcinogenesis.

For many decades, cancer has been thought as a disease resulting from DNA damage. More than often, these resulting DNA products are aberrantly overexpressed or deleted in an individual to promote the growth of cancer cells. Although these protein-coding genes have been widely characterized for their role in tumorigenesis, these DNA regions represent only 2% of the human genome. A large proportion is non-coding and its expression and function have been forgotten until the discovery of non-coding RNAs.

The term non-coding RNA (ncRNAs) is commonly associated with RNA which is not translated into a protein. Indeed many ncRNAs are now understood to have important biological regulatory functions which regulate gene expression at multiple steps/levels^1^. NcRNAs can be divided into two groups encompassing the small and long ncRNA families. The small RNA contingent is approximately 18–29 nucleotides in length and common members include small interfering RNAs (siRNAs), microRNAs (miRNAs) and PIWI associated RNAs (piRNAs).

The lncRNAs can range from several hundred to kilobase size species and evidence now suggests that large non-coding regions of the human genome are transcribed during normal and diseased cellular function^2^,^3^,^4^. These ncRNAs are regulated by distinct promoters, display dynamic spatial temporal expression and regulate protein coding genes central to development and oncogenesis^5^,^6^. In addition, the expression of lncRNAs is dysregulated in many cancers such as breast, colon, liver and also prostate cancer^7^.

Prostate carcinoma represents one of the biggest challenges to the scientific and clinical community as it remains the most common malignancy in men in the western world, where it is still the second leading cause of cancer death^8^. Even though prostate cancer remains a worldwide problem, the exact mechanisms orchestrating the development and progression of prostate cancer are complex and ill defined.

Due to the heterogeneous nature of the disease, this has impeded the discovery of effective clinical markers and the development of novel therapies. The majority of studies to understand this disease have focused on proteins, mRNAs and miRNAs. However, there is limited data on the characterization of the lncRNAs and their role in prostate cancer.

One of the first lncRNAs described in prostate cancer was the prostate cancer antigen 3 (PCA3)^9^. This lncRNA was over expressed in the tumor areas when compared to adjacent normal prostate tissue. However the exact function of PCA3 remains to be determined.

More recently, exosomes^10^ have become important factors in our understanding of tumourigensis^11^. These microvesicles typically 50–150 nm in size are released into the extracellular environment to facilitate communication between cells. Despite their small size, exosomes are enriched in bioactive molecules such as RNA, miRNAs and proteins. It has been demonstrated that tumour derived exosomes shuttle RNA to cells within the tumor environment to promote tumor growth and dampen the immune response^12^.

Our study examines the expression of lncRNAs in several prostate cancer cell lines but also measures the levels of these lncRNAs in the released exosomes. We show that specific lncRNAs are enriched in cancer exosomes and furthermore these sequences harbour miRNA seed regions and appear to be enriched for specific RNA binding motifs.

## Results

### Verification of prostate cancer exosomes

**Figure 1:**
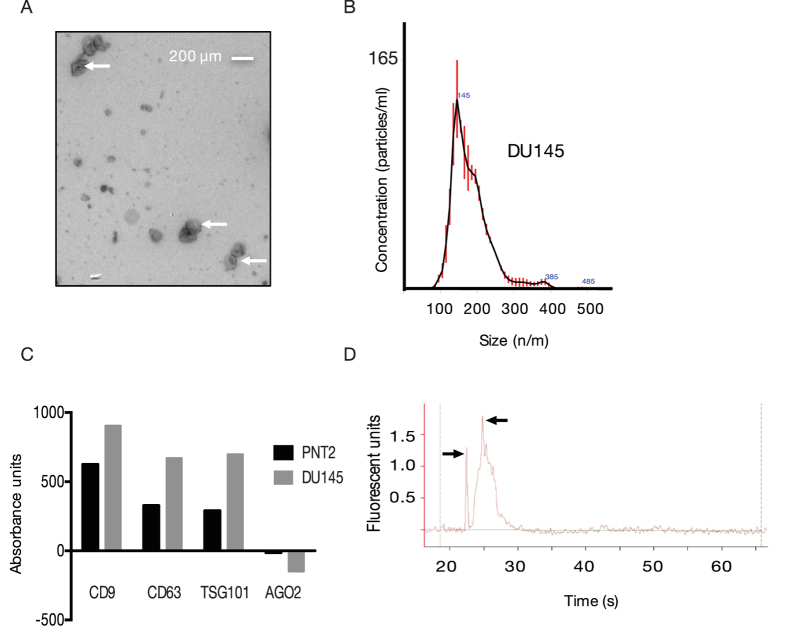
Characterisation of exosomes from *in vitro* cell culture. (**A**) Representative TEM image of DU145 exosomes at 7900 magnifications. White arrows depict exosomes of ~100 μm with kidney bean like appearances. (**B**) Representative particle tracking of a DU145 exosomes using the NanoSight Instrumentation. (**C**) Detection of exosome surface markers, CD9, CD63, TSG101 and AGO2 in DU145 and PNT2 cells. Absorbance was measured by the release of europium using a modified ELISA^36^. (**D**) Small RNA bioanalyser chip image showing the presences of small ncRNAs between 21 and 27 nts (black arrows).

This study used four common prostate cancer cell lines (PC3, VCaP, LNCaP, DU145) and one normal epithelial line (PNT2) to characterize the expression of exosomal lncRNAs. We isolated these exosomes using ultracentrifugation^13^ and then verified these exosomes using Transmission EM, nanoparticle tracking followed by detection of specific transmembrane proteins CD9, CD63, TGS101 and intracellular AGO2 with the Europium assay (Fig. 1)^14^. Transmission EM for one of the representative prostate cancer line DU145 show exosomes ranging from 100–150 nm in size. Nanoparticle tracking of these vesicles indicates that the majority of particles are 145 nm. We then measure the presence of several proteins, which are commonly enriched in exosomes. For exosomes derived from DU145 and the normal cell line PNT2, CD9, CD63, and TGS101 were detected. The AGO2 protein is typically not found or under-represented in exosomes and this was not detected in our isolated exosomes.

### Long ncRNAs are found in prostate cancer exosomes

**Figure 2:**
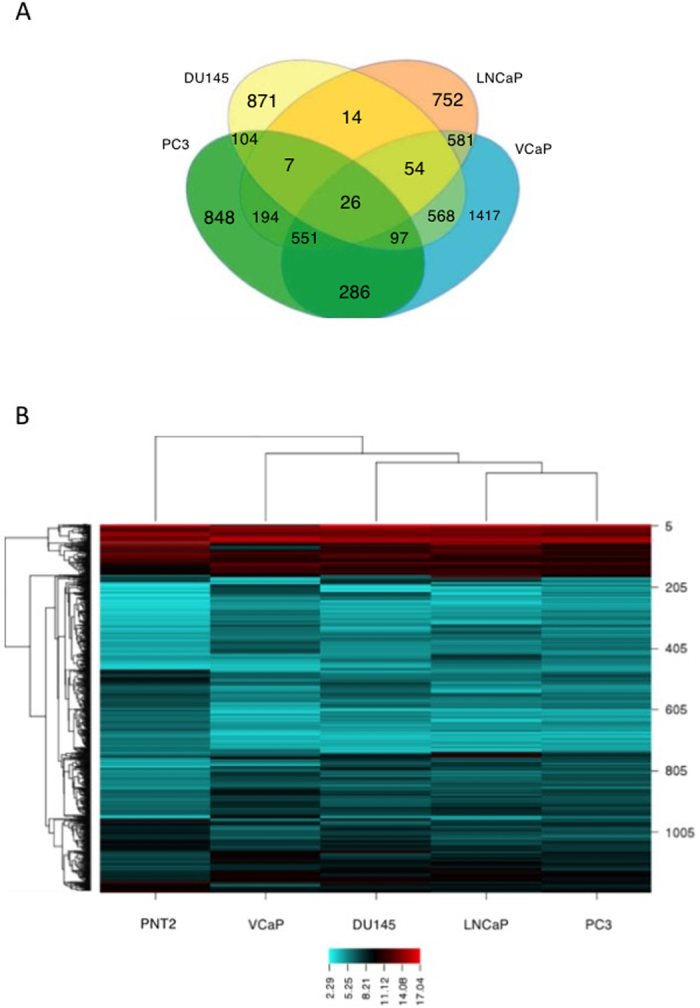
(**A**) Venn diagram showing the common exosomal lncRNAs between the PC3, DU145, LNCaP and VCaP prostate cancer cell lines. (**B**) Heat map showing the expression of exosomal lncRNAs in prostate cancer cells and a normal cell line (PNT2). Red denotes high expression and aqua denotes reduced expression of the lncRNAs.

LncRNA expression was then examined in these exosomes and their parent cell lines. This was performed using a Human 8 × 60 K LncRNA expression array (ArrayStar, Rockville, USA) which contained 33,045 LncRNAs and 30,215 coding transcripts. Results from the array suggested that hundreds of lncRNAs are readily found in exosomes released from prostate cancer cells. We applied a two-fold threshold for expression to identify the most abundant and common lncRNAs in these exosomes and their parent cells (Tables 1 and 2 respectively). The Venn diagram shows that 26 lncRNAs enriched and common to these prostate cancer exosomes (Fig. 2A).

Hierarchical clustering (HCL) analysis of the exosomal lncRNAs indicated that the populations of these lncRNAs could discriminate a normal cell line (PNT2) from a prostate cancer cells (Fig. 2B). The result may suggest that exosomes harbor unique populations of lncRNAs which represent the physiological conditioning of the cell, i.e. diseased versus a normal phenotype.

### miRNA expression profiles in prostate cancer exosomes and cells

**Figure 3:**
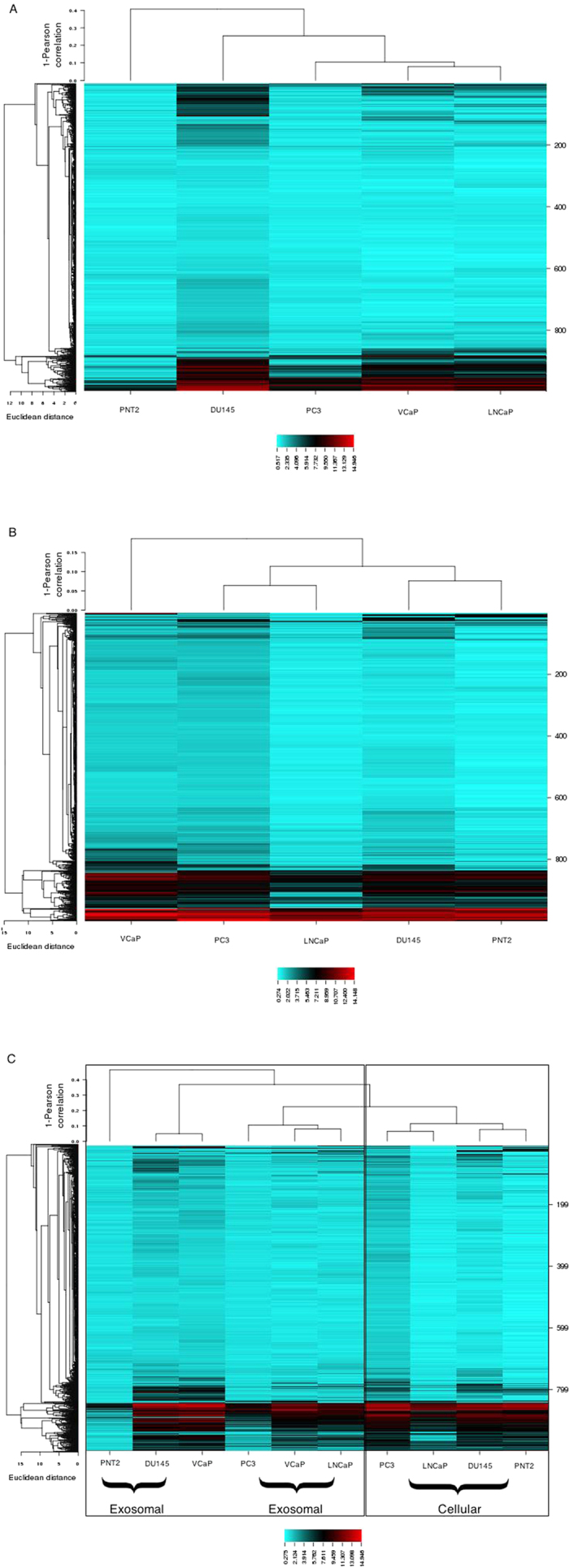
(**A**) miRNA heatmap in exosomes representing prostate cancer and PNT2 normal cell lines. Aqua colour represents reduced levels and Red is indicative of high expression. (**B**) Differential expression of miRNAs in the parental five cell lines. (**C**) Heatmap showing the expression of miRNA in both exosomes and their parental cell line.

We furthered examined the miRNA expression in both exosomes and cells in prostate cancer cells and healthy cells using an Affymetrix miRNA microarray GeneChip® platform. Using Euclidean metrics for HCL analysis, clustering was initially performed on the exosomal data alone, followed by the analysis on the cellular data and lastly combining the two data sets. In the first analysis, the exosomal miRNAs could separate out the four different prostate cancer cell lines from the normal PNT2 line. The VCaP and LNCaP cells branch off a common node, which suggest they may have similar populations of exosomal miRNAs. In contrast, the DU145 and PC3 are unrelated (Fig. 3A). Whereas the clustering pattern in the cellular miRNAs suggests that DU145 shares some commonality with the PNT2 cells. However PNT2 is still distinct when compared to the other three cancer cell lines (Fig. 3B).

When combining the two data sets there are two major clusters, exosomes and cells. The cellular signature of PNT2, DU145, LNCaP and PC3 are distinct from their exosomal miRNAs (Fig. 3C). Furthermore, Pearson’s correlation coefficients indicated that the miRNA expression is highly correlated between cancer cells and exosome (Table 3). This correlation was considerably lower for the normal cells.

### Specific prostate cancer cells contain exosomal lncRNAs which are enriched with different miRNA seed regions

To further understand the role of these exosomal lncRNAs, we hypothesized that they harbored miRNA seed regions as to sequester and bind mature miRNA sequences. To identify these motifs, we performed the enrichment analysis for each of the four cell lines. The data was filtered before analysis and only included transcripts, which were highly enriched in the exosome of each cell line. Several motifs were identified and then aligned to known miRNA sequences. Any motifs, which did not show perfect seed region alignment, were discarded from further analysis.

By using the miRNA expression data, we then measured the miRNA levels in these prostate cancer exosomes and identified the extreme fold change in expression values for these miRNAs in each cell line. In the next step, these motifs were then mapped to the miRNAs showing the highest fold change to identify a perfect seed region alignment. Interestingly, most of highly enriched motifs in each of the cell lines are aligned to at least one miRNA which is highly represented in the exosome of the same cell line. For example, in VCaP, out of the 31 identified highly enriched motifs, 14 of them showed a perfect seed match to at least one miRNA which was also highly elevated in the exosome (Table 4). In the VCaP cell exosomes, the first eight identified motifs have a perfect binding site for the seed match of twenty highly expressed miRNAs. These correspond to *let-7a, let-7b* and *let-7c*, which are the top 3 highly overrepresented miRNAs found in the VCaP exosomes.

We also observe a similar phenomenon in the PC3 cell line. From the 28 identified motifs, eight of them show perfect reverse complementary binding site of 9 highly overexpressed miRNAs. This includes miR-221-5p, miR-762, miR-30b, miR-30c, miR-30d, miR-185, miR-151-5p, miR-130b and miR-149* (Supplementary Table S1). In the LNCaP exosomes, the 28 most highly expressed miRNAs had complementary seed regions, which were enriched in the exosomal lncRNAs. Our analysis identified 18 motifs with U rich sequences and interestingly, a large number of these motifs showed perfect seed region binding sites to miRNAs belonging to two families. These included let-7 family members; *let-7a, let-7b, let-7c, let-7d, let-7e* and *let-7i* as well as the miR-17 family members including miR-17, miR-18a, miR-20a, miR-93 and miR-106b. Upon closer inspection, we observed that all the above-mentioned miRNAs have a perfect seed region match to the motif UGUUUU which is one of the most highly enriched motifs found on these exosomal lncRNAs.

When we extended our enrichment analysis to the normal PNT2 line, we identified 40 over represented motifs in the exosomal lncRNAs. Of these, only three motifs had seed regions to six miRNAs as well as the *let-7* family. Concomitantly these miRNAs were also over expressed in the exosomes. Only one motif, AGCUGG was unique to PNT2 whose seed region is complementary to miR-149-5p. Although these motifs were not entirely unique to PNT2, we did observe that, exosomal lncRNAs only from cancer cells had a higher chance (3–9 fold increase) for seed matching.

These results suggest that lncRNAs, which are enriched in cancer exosomes, also harbor miRNA seed regions and there is a concomitant high representation of that same miRNA in the exosome. Furthermore we observed that *let-7* family and miR-17 families could be associated to U rich motif overrepresented in the lncRNA transcripts expressed in the exosomes.

### miR-149* seed match is highly enriched in prostate cancer exosomes

**Figure 4:**
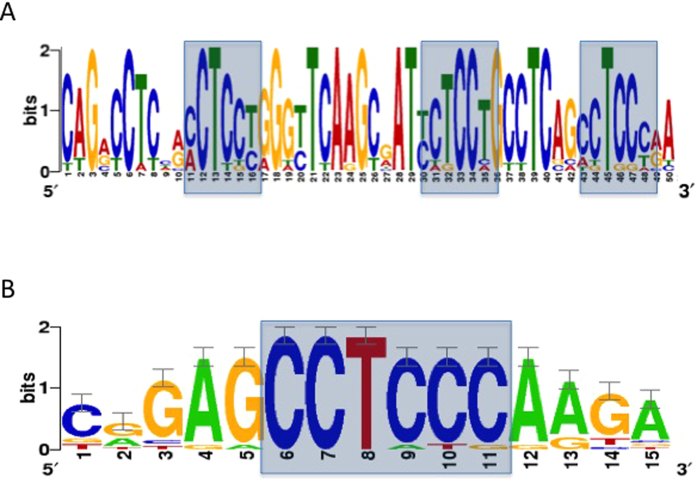
Gapped local alignments of motifs in (**A**) exosomal lncRNAs. (**B**) exosomal lncRNAs vs cellular lncRNAs (control).

From our analysis, (Supplementary Table S2), the motif CCUCCC has the most frequency in the exosomal lncRNA transcripts (Fig. 4A). This motif is a perfect seed match for ten human miRNAs; miR-7106-5p, miR-6883-5p, miR-6799-5p, miR-6785-5p, miR-4728-5p, miR-6887-5p, miR-6885-5p, miR-6799-5p, miR-328-5p and miR-149*.

Out of these ten miRNAs, the only miRNA which we had the expression data available was for miR-149*. When we examined the levels of miR-149* in the exosomes, we realized that the level of this miRNA is significantly (p value = 0.001) high in DU145, LNCaP, PC3 and VCaP with 4.52, 4.03, 3.28 and 1.5 fold change difference respectively (This fold change was the level of miR-149* in the exosome relative to its parent cell). Our observation may suggest that the enrichment of miR-149* seed match motif found in the exosomal lncRNAs is directly related to the high level of miR-149* also found in the exosomes of the prostate cancer cells. To demonstrate the motif is unique to exosomal lncRNAs only, we performed an additional enrichment analysis on exosomal lncRNAs compared to the cellular lncRNAs (control set, Table 2) and identified the same motif (Fig. 4B). We then performed the same analysis on the normal prostate cell line and did not find this motif. Thus it is missing in the transcripts of exosomal lncRNAs of PNT2 cells.

### Prostate cancer cells harbor common motifs in their exosomal lncRNAs

Our next analysis was to identify motifs, which are highly abundant in the common exosomal lncRNAs between all four prostate cancer cell lines compared to common cellular transcripts of the same four cell lines. This search yielded 40 motifs in the exosomal lncRNAs, which harbored a perfect reverse complementary seed match to 697 miRNAs (Supplementary Data S3). Of these motifs, 33 are unique to prostate cancer cells when compared to the PNT2 line. We adopted the same approach for the common lncRNA cellular transcripts and identified only one motif with the sequence of GGGCGC. Interestingly, this motif was not enriched in any of the exosomal lncRNAs from either the cancer or normal cells.

### miRNA seed region enrichment in the exosomal lncRNA is independent of sequence length

To investigate if sequence length was a determinant in miRNA seed enrichment, we compared the average length of all exosomal lncRNAs to the cellular lncRNAs in each cell line. Performing one way ANOVA test, we observed that lncRNAs in the exosomes of VCaP and LNCaP cells were significantly (p value = 0.001) longer than the average length of lncRNAs found inside their parent cells. Further, the DU145 cells did not show a significant mean difference between the length of exosomal and cellular transcripts (p value = 0.28), even though the average length of exosomal lncRNAs is 150 bp longer than the average length of its’ cellular transcripts. In contrast, in the PC3 cells, the mean length of exosomal lncRNAs was considerably less (570 bp difference) than the mean length of cellular lncRNAs. Performing Kendall’s tau-b two-tailed test in SPSS statistical tool, we found no strong correlation between lncRNA sequence lengths and miRNA seed enrichment (Table 5). These analyses suggest that miRNA seed enrichment in the exosomal lncRNAs was independent of sequence length and may suggest the enrichment is linked to exosomal transport of the lncRNAs from the cell.

### Exosomal lncRNAs are enriched with specific RBP binding sites

**Figure 5:**
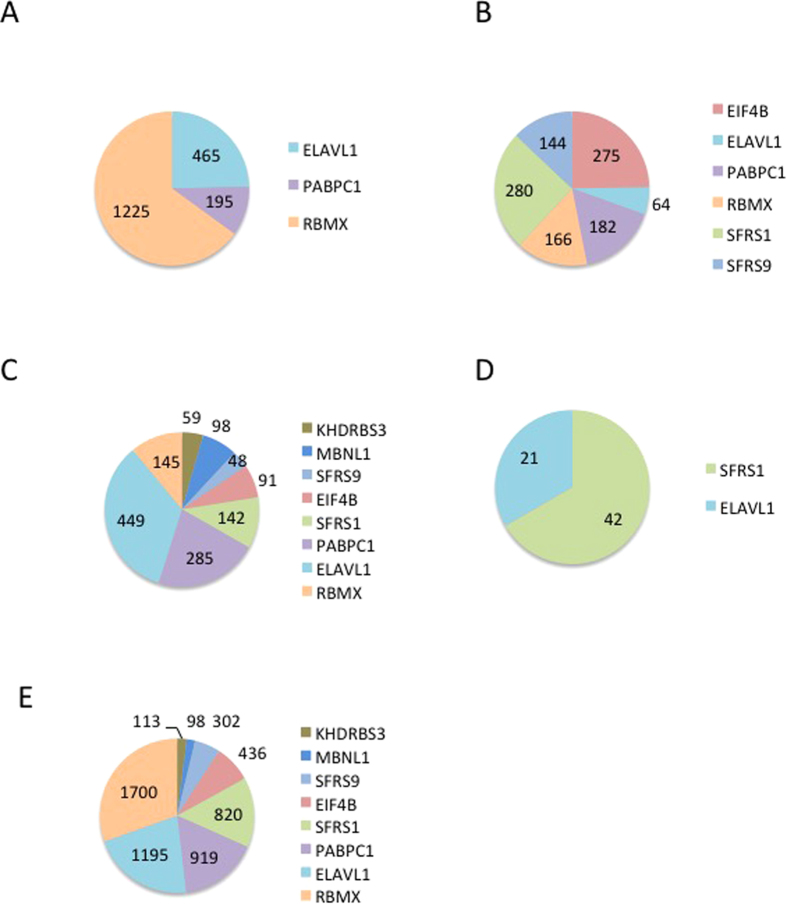
The number of positive matching RBP binding sites found in the exosomal lncRNAs for each cell line (**A**) VCaP, (**B**) PC3, (**C**) LNCaP, (**D**) DU145, (**E**) Distribution of RNA binding sites in exosomal lncRNAs of at least one cancer cell line.

We then sought to investigate if there existed short motifs, which could associate with RNA binding proteins. Our first analysis was restricted to the exosomal lncRNAs. We performed RBP binding site enrichment analysis on different sets of exosomal lncRNAs transcripts including lncRNAs which are enriched in all prostate cancer cell lines (N = 26), lncRNAs which are enriched in the exosome of healthy prostate cells (N = 32), and the set of lncRNAs which are enriched in the cell of at least one prostate cancer cell line (N = 141, Supplementary Table S5), and the exosomal lncRNAs enriched in each prostate cancer cell line. Towards this end, we aligned the genomic sequence of these exosomal lncRNAs to the RNA binding protein database (RBPDB)^15^. This database is a collection of 424 experimentally validated human RNA-binding sites collected from literature, which includes 73 position-based matrix (PWM). We used the RBPDB default value 0.8% as the minimum threshold score for any matches between identified motifs in our given sequences and RNA-binding sequence in the database. We identified a total number of 38 RBPs with confident binding sites (Supplementary Data S4). Each of these RBPs has a different number of binding sites on the exosomal lncRNAs. However, RBMX, SFRS1 and ELAVL1 have the highest number of confident binding sites. To confirm this finding using our algorithm, we identified 33, 42, 63 and 28 motifs (six bases in size), which were highly enriched in VCaP, PC3, LNCaP and DU145 cells respectively (Supplementary Data S6). These short nucleotide sequences represented 126 distinct motifs which from 43 are binding sites for at least one of the following RBPs: EIF4B, ELAVL1, KHDRBS3, MBNL1, PABPC1, SFRS1, SFRS9 and RBMX. The number of positive matching RBP binding sites found in the exosomal lncRNAs for cancer cell line is shown in Fig. 5A–D. Our analysis shows that there is a clear over representation of motifs for ELAVL1, PABPC1, SFRS1 and RBMX (Fig. 5E). Using our algorithm, we also observed the over-representation of the binding sites of RBMX, SFRS1 and ELAVL1 in the set of enriched exosomal lncRNAs in cancer cell lines and in the healthy cells.

### Comparative RBP site enrichment analysis in exosomal lncRNAs and cellular lncRNAs

**Figure 6:**
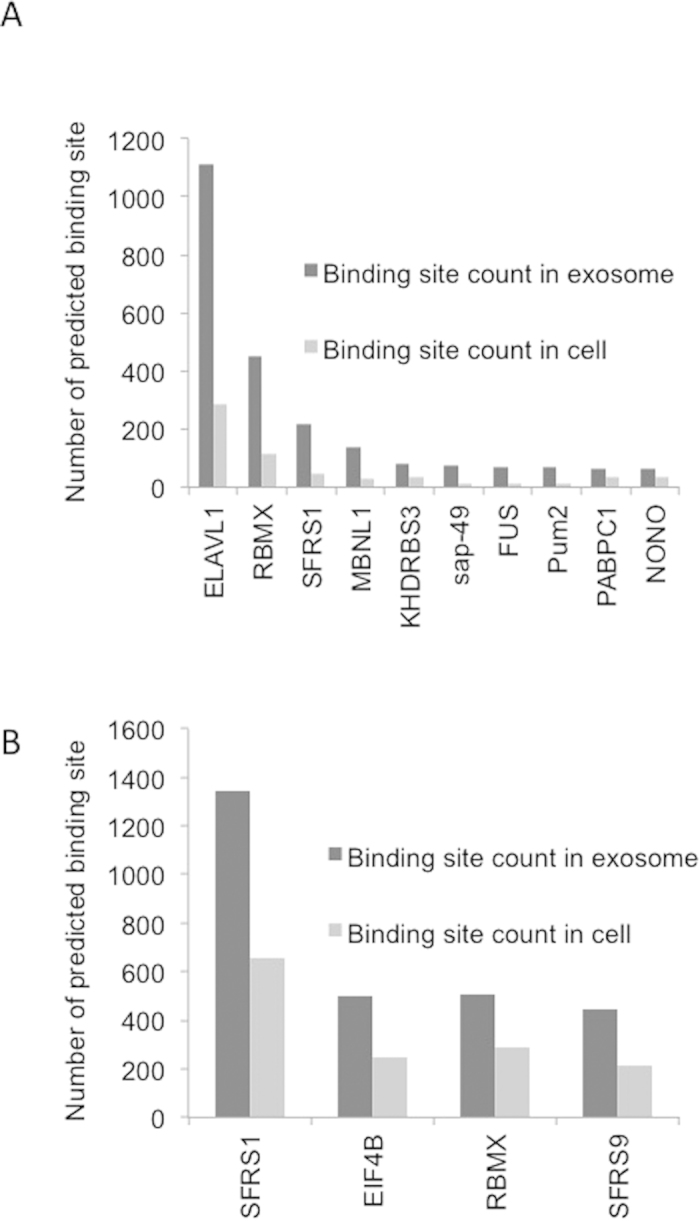
Comparison of RNA binding motifs on exosomal lncRNAs versus cellular transcripts. (**A**) Reduction of RBP motifs in cellular lncRNAs which are common to all four cell lines. In contrast there is a increase of these motifs in the exosomal lncRNAs. (**B**) Increase in RBP motifs of the enriched exosomal lncRNAs (141 transcripts) to those lncRNAs, which were highly expressed in the cells (151 transcripts) of at least one cell line. This analysis yielded four major binding sites for, RBMX, SFRS1, SFRS9 and EIF4B.

Using our algorithm, we then examined if the over-represented motifs in the exosomal transcripts compared to cellular transcripts shows a significant reduction in the binding site availability for RBPs. Using a set of 19 lncRNAs which were common to all cancer cell lines but not enriched in exosomes (Table 2), we found a significant reduction (p value = 0.001) in enriched binding sites for both RBMX and ELAVL1 (Fig. 6A). We then extended this analysis by comparing lncRNAs which were enriched in the exosome (141 transcripts) to those lncRNAs which were highly expressed in the cells (151 transcripts) of at least one cancer cell line (Supplementary Table S5). This comparative analysis identified binding sites for four distinct RBPs, RBMX, SFRS1, SFRS9 and EIF4B. However there is a ~50% reduction in the frequency of these binding sites for the cellular lncRNAs (Fig. 6B).

Our analysis on the enrichment of healthy cells also shows significant reduction of the binding site of these RBPs in the highly enriched lncRNAs of exosomes compared to the ones in the cells. We did not identify any RBP which uniquely target either the exosomal or the cellular lncRNAs, however there is a significant difference in the number of binding sites for RBMX, ELAVL1 and SFRS1 in the exosomal lncRNAs compared to cellular lncRNAs of both healthy and cancer cell lines.

## Discussion

This study measured the levels of long non-coding RNAs in exosomes released from prostate cancer cells and healthy cells. The four prostate cancer cell lines represent common metastatic lines isolated from different tissue sites. They have different metastatic potential and are either hormone sensitive/resistant. The array data indicated that lncRNAs are present in abundance in both healthy and prostate cancer exosomes. Furthermore, certain lncRNAs are specifically enriched only in the exosomes. When we applied HCL analysis, these exosomal lncRNAs were able to cluster the normal cell (PNT2) from the cancer cell lines. More so, LNCaP and PC3 have similar expression when compared to the DU145 and VCaP cells. We put forward the idea that exosomal lncRNAs signatures may represent a unique set of ncRNAs, which can be exploited for biomarker research. There have been a plethora of studies suggesting lncRNAs as biomarkers for prostate cancer, but only a few studies have characterized their presence in exosomes^16^,^17^. This is one of the few studies to catalogue the many thousands of exosomal lncRNAs in prostate cancer cells with a comparative analysis to normal epithelia cells.

Several studies have suggested that lncRNAs can act as sponges for miRNAs^18^,^19^. We extended this idea and sought to investigate if these exosomal lncRNAs contained any miRNA seed regions. Firstly, we identified enrich motifs in the exosomal lncRNAs and then mapped these motifs to only highly expressed miRNAs found in the same exosomes. We found that most of the enriched motifs were perfectly aligned to (seed regions) of at least one exosomal miRNA from the same parent cell line. In the VCaP exosomes, there was seed enrichment in their lncRNAs for the *let-7* family members. While in the LNCaP exosomes seeds sequences for *let-7* and miR-17 members were highly represented on the exosomal lncRNAs. Another study demonstrated that *let-7* was highly expressed in the blood of prostate cancer patients and also enriched in the exosomes of PC3 cells^20^. Furthermore there is now evidence that *let-7* can be selectively package into exosomes derived from metastatic gastric cancer cell lines^21^.

Upon further analysis, we found 40 motifs with perfect miRNA seed regions, which were highly represented in the exosomal lncRNAs. In contrast only a solitary motif was identified in the cellular lncRNAs. In particular, CCUCCC was highly abundant with this region corresponding to the miR-149* seed. Interestingly, miRNA expression data indicated that miR-149* was significantly (p value = 0.001) elevated only in the exosomes and not the cell lines. The apparent enrichment of seed regions may suggest a possible role for these exosomal lncRNAs as RNA sponges for specific miRNAs.

RNA-binding proteins (RBPs) are important regulators of many post-transcriptional events, including RNA splicing, transport and stability. The RBP ELAVL1 (also known as HuR) tends to stabilize RNA transcripts; while AUF1, may induce rapid degradation of RNA transcripts^22^. In our analysis we identified several RBP motifs on these lncRNAs. The two most common motifs were associated with ELAVL1 and RBMX. These is a ~ two-fold increase for these sites in exosomal lncRNAs when compared to cellular lncRNAs. Furthermore we did not find any unique sites from our comparative analysis.

ELAVL1 is a member of the ELAVL family of RBP which posses RNA recognition motifs to selectively bind AU-rich elements (AREs) in the 3′ UTR regions of mRNAs. Binding of these AREs by ELAVL1 prevents the signalling events required for degradation thus stabilizing the mRNA transcript. It may be possible that ELAVL1 can extend its function to bind lncRNAs with abundant AREs to stabilize the transcript. Evidence for this scenario was documented when the lncRNA-UFC1 could directly interact with ELAVL1 in hepatocellular carcinoma cells^23^. ELAVL1 can also modulate transcriptome-wide miRNA binding to target RNAs in murine macrophages^24^. It was shown that transcripts bearing ELAVL1 sites proximal to a miRNA site demonstrated attenuated miRNA binding. There appears to be a complex interplay between miRNA binding and ELAVL1 for regulating RNA expression^25^,^26^,^27^.

RBMX is a ubiquitous nuclear ribonucleoprotein which interacts with the spliceosome, binds RNA, and is involved in pre-mRNA splicing^28^. It most likely binds RNA as a homodimer and is preferential to single-stranded 5′-CC[A/C]-rich RNA motifs. RBMX has been shown to be present in both micro particles and exosomes isolated from endothelial and ovarian cancer cells^29^,^30^. A study has shown that RBMX regulates the release of TNFR1 exosome-like vesicles into the extracellular matrix^31^. Their binding capacity to exosomal lncRNAs has not been postulated, however we predict that RBMX may mark the lncRNA into exosomes for transport akin to its function for TNFR1.

It is important to also recognize the caveats in our study. The cell lines were grown *in vitro* conditions to a confluence of 70–80% with the rationale that, it would reflect fast growing cancer cells *in vivo*. We have avoided 100% confluence, as cells would likely exit this exponential growth phase. It has been shown that altering the growth conditions *in vitro* can significantly change the cellular miRNA composition^32^. With this in mind, we put forward the notion that lncRNAs along with specific RBPs act to corral these miRNAs into exosomes. Given that most lncRNAs harbor both seed match regions and RBP binding sites, it may be a general mechanism for shuttling miRNAs into exosomes.

In summary, our study has catalogued the presence of lncRNAs in exosomes from prostate cancer and normal cell lines. These exosomal lncRNAs seem to be enriched in exosomes and harbor both miRNA and RBP binding sites. The populations of exosomal lncRNAs as indicated by HCL derived from prostate cancer cells are distinct from normal cells. This suggests that exosomal lncRNAs are disease specific and may provide a source of biomarkers for prostate cancer diagnosis.

The mechanism for loading exosomal lncRNAs into exosomes is currently unknown. Given the enrichment of distinct RBP motifs on exosomal lncRNAs, we predict that specific RBPs such as ELAVL1 may play an important role in directing lncRNAs for exosomal transport. Many of these exosomal lncRNAs also harbored miRNA binding regions with an over representation for specific miRNA families such as *let-7*. Several lncRNAs have been shown to have a sponge effect^33^,^34^ and similarly these exosomal lncRNAs may partake in this regulation but with the added effect of transporting these miRNA into exosomes and eventual release into the extracellular milieu.

The fact that exosomal lncRNAs have both an enrichment of miRNA and RBP does elute to a possible function in prostate cancer cells. However, further exploration will be needed to fully understand the interplay between these exosomal lncRNAs, miRNAs and RBPs.

## Methods

### Cell lines

The prostate cancer cell lines, LNCaP, PC3, and DU145 were grown in RPMI, whereas VCaP cells were grown in F12:DMEM and PNT2 in defined KSFM (Invitrogen, USA). All cells were incubated at 37 °C in 5% CO_2_, and supplemented with 1% v/v penicillin, streptomycin, glutamine (PSG) and 10% v/v fetal calf serum (FCS).

### Exosome production and purification

As FCS contains exosomes of bovine origin all media were first depleted of these bovine exosomes. To remove bovine exosomes, we utilized the ultracentrifugation protocol devised by Théry, C. *et al*.^35^. First, media was constituted with all the supplements and centrifuged at 104 492 × g (28 000 rpm using F40L-8 × 100 rotor (Thermo Scientific, USA)) overnight at 4 °C. Media was then filtered through a 0.2 μm filter and a further 500 mL of basic media supplemented with PSG was added. Depleted media was stored at 4 °C for no longer than four weeks. For exosome production, cells were seeded in 175 cm^2^ flasks containing 25 mL of depleted media at an initial confluence of 15–20% and allowed to grow to 70–80% confluence, at which point the culture supernatant and cells were harvested. Once culture supernatant was harvested it was either stored at 4 °C for one to five days prior to exosome extraction by ultracentrifugation or frozen at −80 °C and thawed the day before extraction. All exosome preparations were performed according to the protocol described by Théry *et al*.^35^ which is the current “gold standard” for exosome extraction. Confirmation of exosomes was performed as previously described^14^,^36^.

### RNA extraction and long ncRNA arrays

RNAzol RT (Molecular Research Center, USA) was used to extract total RNA from both cellular and exosomal sources. Given the small size pellet for the exosomes we had to modify the manufacture’s protocol. In brief, 500 μL aliquot of RNAzol RT was used to resuspend the exosome pellet. The same mixture was then transferred to a new pellet and resuspended. This method was used to concentrate the total RNA from multiple exosome pellets. Total RNA isolation was then completed using the manufacture’s protocol with the addition of glycogen (25 μg) as a carrier and an overnight precipitation step. Total RNA was isolated from cells using the manufacture’s protocol. RNA quality was the assessed using an Agilent 2100 bioanalyser and small RNA chip (Agilent Technologies, USA). Samples that reached the 50 ng/μL cut-off, and a RIN value of 8–10 were subjected to lncRNA expression profiling. Total RNA from the exosomes and their parent cell were subjected to lncRNA profiling using the Human 8 × 60 K LncRNA expression array (ArrayStar, Rockville, USA). Each array contained 33,045 LncRNAs and 30,215 coding transcripts and required a minimum of 2 μg of total RNA.

### Pipeline of enriched motif identification

The algorithm used in this study takes two sets of sequences including exosomal (test) and cellular (control) transcripts as input and identifies a set of 6-mers which are a) highly enriched in exosomal transcripts compared to the cellular transcripts and b) harbor the reverse complementary match of the seed region of at least one known miRNA. For each set, a sliding window of length six scans the whole length of all given sequences and extracts all possible motifs of length six. The reason we selected the length of six nucleotides is for the importance of the miRNA seed region match in governing miRNA binding to the target transcript. Next, all 6-mers of each set with a frequency occurrence of one are discarded. Among identified 6-mers, those motifs which have an equal occurrence frequency in both sets are discarded. At this stage, we performed motif enrichment analysis on the sequences of each set using MEME^37^ and RSAT peak-motifs^38^ tools to validate the output of our approach. For all investigated sets of sequences in this study, we made sure that all discovered motifs of recently mentioned tools are indeed identified by our approach as well.

Next, among all identified motifs in each set, all motifs which show a perfect reverse complementary Watson-Crick match to the seed region of at least one miRNA annotated in the miRBase v20^39^ were identified. This includes 2578 distinct mature miRNA sequences. Those 6-mers which do not show a perfect match to the seed region of any miRNA are discarded. In seed match identification, G:U wobbles were allowed, but no bulge, gap or mismatch were allowed. Among all motifs identified in the sequence of exosomal transcripts, those which the occurrence frequency value in the exosomal set is greater than occurrence frequency value in cellular set were identified. The result of the last stage provide a list of short motifs harboring miRNA seed match regions in each set which are enriched in exosomal transcripts but are not abundant in the sequence of cellular transcripts. In the end, z score values associated to occurrence frequency difference distribution of identified motifs was calculated and a cut off value of three was used to identify those motifs which are extremely enriched in the sequence of exosomal transcripts compared to the cellular transcripts with a confidence level of 0.999. At this stage, we performed comparative motif enrichment analysis using the sequences of both sets using DREME^40^ and RSAT tools to validate the final output of our approach. For all investigated exosomal sets of sequences in this study, we made sure that all discovered enriched motifs in exosomal transcripts compared to the cellular transcripts (control) identified by DREME tool are indeed identified by our approach as well. Comparative enrichment analysis between two sets of genomic sequences might be affected by the length of the transcripts in each set. Generally speaking, a long transcript might have a higher occurrence frequency of a given motif in comparison to a control sequence with shuffled nucleotides. Since the comparative enrichment analysis of motifs between the exosomal transcript and cellular transcripts is a part of this study, we investigated the variance in lengths of input sequences for two sets, i.e. exosomal and cellular transcripts, is not misleading the outcome of the algorithm. We performed correlation analysis between the length of each transcript and the occurrence frequency of each enriched motif in it using Kendall’s tau-b correlation test which is suitable for non-normally distributed bivariate data. We performed Kendall’s tau-b two tailed test on four different populations of selected transcripts. As could be seen in Table 5, the coefficient value of the investigated correlations for both cellular and exosomal transcripts indicate a weak correlation between the length of the transcripts and the motif enrichment. We avoided using Pearson and Spearman correlation analysis for two main reasons. Firstly, both these techniques are slightly sensitive to outliers^41^ and the presence of extreme values can have great influence on their correlation coefficients. Secondly, these tests are not appropriate for highly skewed distributions which is the case here.

### Data Preparation

Genomic sequences for transcripts were obtained from Ensembl or NCBI nucleotide database according to their accession IDs. For those transcripts for which the genomic sequence was not available on any publicly available database, the genomic sequence was prepared in BioPython. All poly-Adenylation tails were removed from the sequences in order to optimize the result of the algorithm. MicroRNA and lncRNA expression Heat maps were designed using CIMminer (http://discover.nci.nih.gov). The enrichment logo was designed using WebLogo^42^. The gapped enrichment sequence was designed using GLAM2^43^.

In order to find exosome specific lncRNAs, up-regulated transcripts with a fold change value greater than two in each cell line were selected. Identification of over-expressed transcripts in each cell line was performed by re-scaling of the expression level of each transcript using z-score transformation as described in^44^. The cut off value of three for z score was used to select transcripts which have an extreme expression value with a confidence level of 0.999. Over expressed miRNAs of each cell line were identified using SPSS outlier detection. In order to perform differential expression analysis of miRNAs in cells versus exosomes as well as correlation analysis of differentially expressed miRNAs, all miRNA expression values were rescaled to log_10_ fold change.

## Additional Information

**Data Availability**: MicroRNA expression, lncRNA expression and mRNA expression data in all prostate cancer cell lines investigated in this study are available on request.

**How to cite this article**: Ahadi, A. *et al*. Long non-coding RNAs harboring miRNA seed regions are enriched in prostate cancer exosomes. *Sci. Rep.* **6**, 24922; doi: 10.1038/srep24922 (2016).

## Supplementary Material

Supplementary Tables

Supplementary Data 3

Supplementary Data 4

Supplementary Data 6

## Figures and Tables

**Table 1:** Common lncRNAs which are enriched in the exosomes of all four prostate cancer cell lines (VCaP, LNCaP, DU145 and PC3).

Gene name	VCaP	LNCaP	DU145	PC3	Long ncRNA type
ENST00000501280	62.0	21.4	21.4	14.7	bidirectional
uc010bys.1	28.7	11.0	11.0	14.6	intergenic
uc001qgn.1	50.0	5.4	5.4	3.0	intronic antisense
ENST00000499690	15.4	16.2	16.2	8.5	intronic antisense
ENST00000453968	48.5	2.5	2.5	2.3	intergenic
G36642	46.0	3.0	3.0	2.2	intronic antisense
AK055500	39.1	3.5	3.5	6.1	intergenic
HIT000070262	44.2	2.7	2.7	2.1	natural antisense
ENST00000452932	11.8	9.5	9.5	7.7	intergenic
chr13:38754175-38773200+	25.6	3.7	3.7	2.8	intergenic
nc-HOXB1-156-	7.4	8.4	8.4	2.6	intergenic
ENST00000471393	18.5	2.3	2.3	3.5	intergenic
ENST00000412906	20.0	2.2	2.2	2.1	intronic antisense
ENST00000392478	3.7	6.2	6.2	2.0	intronic antisense
AY927529	7.0	2.6	2.6	5.8	exon-sense overlapping
ENST00000426885	5.8	3.3	3.3	4.5	Intergenic
AK123811	2.3	3.7	3.7	4.9	intergenic
ENST00000402340	5.7	2.3	2.3	3.7	intergenic
ENST00000509526	4.9	2.3	2.3	3.4	intergenic
ENST00000412347	4.1	2.5	2.5	3.0	intergenic
chr9:137160729-137173954-	3.8	2.4	2.4	2.8	intergenic
ENST00000411341	3.3	2.3	2.3	3.2	intergenic
ENST00000430245	2.8	2.9	2.9	2.4	intronic antisense
ENST00000508864	3.9	2.4	2.4	2.1	intergenic
uc001upm.2	2.2	2.0	2.0	4.3	intergenic
ENST00000500478	3.2	2.2	2.2	2.3	intergenic

The threshold cut-off was a fold change greater then 2. Fold change was defined as the ratio of normalized intensities between two samples (exosome versus cell), positive value indicates up-regulation.

**Table 2:** LncRNAs which are in common and enriched in the four cell lines.

Gene name	VCaP	LNCaP	DU145	PC3	lncRNA type
AK098134	−48.6	−3.5	−3.5	−14.1	intergenic
uc.63-	−18.6	−3.4	−3.4	−9.6	intron sense-overlapping
uc004exm.2	−7.2	−2.1	−2.1	−22.2	intergenic
ENST00000513351	−23.2	−2.4	−2.4	−4.3	intergenic
BC008292	−10.9	−2.7	−2.7	−2.7	bidirectional
BC131500	−4.1	−5.0	−5.0	−2.0	intronic antisense
AK125090	−6.5	−2.6	−2.6	−3.5	intergenic
ENST00000366160	−3.0	−3.4	−3.4	−5.2	intergenic
NR_002998	−3.5	−2.0	−2.0	−7.2	intron-sense overlapping
G43604	−4.0	−3.2	−3.2	−2.9	natural antisense
NR_024413	−4.1	−2.0	−2.0	−3.9	intron-sense overlapping
uc003hqg.1	−5.3	−2.1	−2.1	−2.4	intronic antisense
uc003eub.2	−3.6	−2.0	−2.0	−4.1	intergenic
NR_024334	−2.7	−3.3	−3.3	−2.5	natural antisense
uc003nov.3	−3.1	−2.6	−2.6	−2.4	intergenic
ENST00000429037	−2.6	−2.4	−2.4	−2.6	intergenic
HIT000097222	−2.7	−2.1	−2.1	−3.3	natural antisense
AK097323	−2.0	−2.7	−2.7	−2.2	natural antisense
NR_002836	−2.2	−2.4	−2.4	−2.3	intergenic

Fold change was defined as above but in this instance, fold change represent (cell versus exosome). Negative values indicate down-regulation.

**Table 3:** Correlation of miRNA expression between the parental cell and the exosome: All correlations are significant at 0.01 level (2-tailed).

Cell line	Pearson’s correlation coefficient of normalized expression fold change	p value
VCaP	0.95	0.0001
PC3	0.93	0.0001
DU145	0.92	0.0001
LNCaP	0.88	0.0001
PNT2	0.70	0.0001

**Table 4:** Overrepresentation of miRNA seed region motifs in highly enriched exosomal lncRNAs from the VCaP cell line.

Motif	miRNA	Fold-change
CAGCCU	hsa-miR-762	10.39
CUCCUG	hsa-miR-30b-3p	8.57
	hsa-miR-1275	10.93
CCUCCC	hsa-miR-149-3p	9.46
AUUUUU	28 distinct miRNAs	NA
GCCUCC	13 distinct miRNAs	NA
CUGCCU	hsa-miR-363-5p	8.54
GCUGGG	hsa-miR-221-5p	7.92
UAUUUU	hsa-miR-20b-5p	11.07
	hsa-miR-106b-5p	11.51
	hsa-miR-17-5p	12.76
	hsa-miR-18a-5p	10.07
	hsa-miR-106a-5p	12.49
	hsa-miR-93-5p	11.98
	hsa-miR-20a-5p	11.85
	hsa-let-7a-5p	13.20
	hsa-let-7b-5p	14.52
	hsa-let-7c-5p	14.16
	hsa-let-7d-5p	12.22
	hsa-let-7e-5p	11.89
	hsa-let-7g-5p	9.29
	hsa-let-7i-5p	10.89
CCCAGC	4 distinct miRNAs	NA
CCAGGC	4 distinct miRNAs	NA
CCUGGG	16 distinct miRNAs	NA
CCCAGG	6 distinct miRNAs	NA
CCUCCU	hsa-miR-149-3p	9.46
CUGGGA	11 distinct miRNAs	NA
GGCUGG	21 distinct miRNAs	NA
CUCCCA	hsa-miR-1275	10.93
	hsa-miR-30b-3p	8.57
AGGCUG	10 distinct miRNAs	NA
CUCCAG	8 distinct miRNAs	NA
CCUCAG	11 distinct miRNAs	NA
UCCCAG	18 distinct miRNAs	NA
UUUUUA	hsa-miR-1275	10.93
	hsa-miR-30b-3p	8.57
CCUGCC	hsa-miR-1207-5p	8.70
GCCCAG	9 distinct miRNAs	NA
UGGGAG	13 distinct miRNAs	NA
GGGAGG	20 distinct miRNAs	NA
CUUCCU	hsa-miR-149-3p	9.46
CUGCAG	11 distinct miRNAs	NA
CUGGGC	hsa-miR-423-3p	9.33
UGCCUC	hsa-let-7g-5p	9.29
	hsa-miR-3196	10.18
	hsa-let-7e-5p	11.89
	hsa-let-7d-5p	12.22
	hsa-let-7c-5p	14.16
	hsa-let-7b-5p	14.52
	hsa-let-7a-5p	13.20
CUCAGC	7 distinct miRNAs	NA
UUUUCU	hsa-miR-185-5p	9.25
	hsa-miR-149-3p	9.46

Fold change of the miRNAs is shown in the last column. Note: All motifs listed in the table represent the perfect reverse complementarity to the seed region of specific miRNAs.

**Table 5:** Kendall’s tau-b two tailed test result for correlation analysis of the length of transcripts and motif enrichment frequency.

Selected transcripts	Number of transcripts	Correlation coefficient	Correlation strength
All exosomal transcripts	26	0.52	weak
All cellular transcripts	19	0.16	negligible
All over expressed transcripts in cancer exosomes	141	0.42	weak
All over expressed transcripts in cancer cells	151	0.24	negligible
All correlations are significant at 0.01 level.			

## References

[b1] Mattick J. S. & Makunin I. V. Non-coding RNA. Hum Mol Genet 15 Spec No 1, R17–29, doi: 10.1093/hmg/ddl046 (2006). 16651366

[b2] Jacquier A. The complex eukaryotic transcriptome: unexpected pervasive transcription and novel small RNAs. Nat Rev Genet 10, 833–844, doi: 10.1038/nrg2683 (2009). 19920851

[b3] Carninci P. . The transcriptional landscape of the mammalian genome. Science 309, 1559–1563, doi: 10.1126/science.1112014 (2005). 16141072

[b4] Johnson J. M., Edwards S., Shoemaker D. & Schadt E. E. Dark matter in the genome: evidence of widespread transcription detected by microarray tiling experiments. Trends Genet 21, 93–102, doi: 10.1016/j.tig.2004.12.009 (2005). 15661355

[b5] Guffanti A. . A transcriptional sketch of a primary human breast cancer by 454 deep sequencing. BMC Genomics 10, 163, doi: 10.1186/1471-2164-10-163 (2009). 19379481 PMC2678161

[b6] Mercer T. R., Dinger M. E. & Mattick J. S. Long non-coding RNAs: insights into functions. Nat Rev Genet 10, 155–159, doi: 10.1038/nrg2521 (2009). 19188922

[b7] Rossi S., Sevignani C., Nnadi S. C., Siracusa L. D. & Calin G. A. Cancer-associated genomic regions (CAGRs) and noncoding RNAs: bioinformatics and therapeutic implications. Mamm Genome 19, 526–540, doi: 10.1007/s00335-008-9119-8 (2008). 18636290

[b8] Parkin D. M., Pisani P. & Ferlay J. Global cancer statistics. CA Cancer J Clin 49, 33–64 (1999). 10200776 10.3322/canjclin.49.1.33

[b9] Lu W. . KLK31P is a novel androgen regulated and transcribed pseudogene of kallikreins that is expressed at lower levels in prostate cancer cells than in normal prostate cells. Prostate 66, 936–944, doi: 10.1002/pros.20382 (2006). 16541416

[b10] Trams E. G., Lauter C. J., Salem N. Jr. & Heine U. Exfoliation of membrane ecto-enzymes in the form of micro-vesicles. Biochim Biophys Acta 645, 63–70 (1981). 6266476 10.1016/0005-2736(81)90512-5

[b11] Zitvogel L. . Dendritic cells or their exosomes are effective biotherapies of cancer. Eur J Cancer 35 Suppl 3, S36–38 (1999). 10645221 10.1016/s0959-8049(99)00090-8

[b12] Keller S. . Systemic presence and tumor-growth promoting effect of ovarian carcinoma released exosomes. Cancer Lett 278, 73–81, doi: 10.1016/j.canlet.2008.12.028 (2009). 19188015

[b13] Thery C., Amigorena S., Raposo G. & Clayton A. Isolation and characterization of exosomes from cell culture supernatants and biological fluids. Curr Protoc Cell Biol Chapter 3, Unit 3 22, doi: 10.1002/0471143030.cb0322s30 (2006). 18228490

[b14] Lotvall J. . Minimal experimental requirements for definition of extracellular vesicles and their functions: a position statement from the International Society for Extracellular Vesicles. Journal of extracellular vesicles 3, 26913, doi: 10.3402/jev.v3.26913 (2014). 25536934 PMC4275645

[b15] Cook K. B., Kazan H., Zuberi K., Morris Q. & Hughes T. R. RBPDB: a database of RNA-binding specificities. Nucleic Acids Res 39, D301–308, doi: 10.1093/nar/gkq1069 (2011). 21036867 PMC3013675

[b16] Gezer U., Tiryakioglu D., Bilgin E., Dalay N. & Holdenrieder S. Androgen Stimulation of PCA3 and miR-141 and Their Release from Prostate Cancer Cells. Cell journal 16, 488–493 (2015). 25685739 10.22074/cellj.2015.494 PMC4297487

[b17] Isin M. . Exosomal lncRNA-p21 levels may help to distinguish prostate cancer from benign disease. Frontiers in genetics 6, 168, doi: 10.3389/fgene.2015.00168 (2015). 25999983 PMC4422020

[b18] Ebert M. S. & Sharp P. A. Emerging roles for natural microRNA sponges. Curr Biol 20, R858–861, doi: 10.1016/j.cub.2010.08.052 (2010). 20937476 PMC4070712

[b19] Wang Y. . Endogenous miRNA sponge lincRNA-RoR regulates Oct4, Nanog, and Sox2 in human embryonic stem cell self-renewal. Dev Cell 25, 69–80, doi: 10.1016/j.devcel.2013.03.002 (2013). 23541921

[b20] Hessvik N. P., Phuyal S., Brech A., Sandvig K. & Llorente A. Profiling of microRNAs in exosomes released from PC-3 prostate cancer cells. Biochim Biophys Acta 1819, 1154–1163, doi: 10.1016/j.bbagrm.2012.08.016 (2012). 22982408

[b21] Ohshima K. . Let-7 microRNA family is selectively secreted into the extracellular environment via exosomes in a metastatic gastric cancer cell line. PLoS One 5, e13247, doi: 10.1371/journal.pone.0013247 (2010). 20949044 PMC2951912

[b22] Gratacos F. M. & Brewer G. The role of AUF1 in regulated mRNA decay. Wiley interdisciplinary reviews. RNA 1, 457–473, doi: 10.1002/wrna.26 (2010). 21956942 PMC3608466

[b23] Cao C. . The long intergenic noncoding RNA UFC1, a target of MicroRNA 34a, interacts with the mRNA stabilizing protein HuR to increase levels of beta-catenin in HCC cells. Gastroenterology 148, 415–426 e418, doi: 10.1053/j.gastro.2014.10.012 (2015). 25449213

[b24] Lu Y. C. . ELAVL1 modulates transcriptome-wide miRNA binding in murine macrophages. Cell reports 9, 2330–2343, doi: 10.1016/j.celrep.2014.11.030 (2014). 25533351 PMC4277505

[b25] Connerty P., Ahadi A. & Hutvagner G. RNA Binding Proteins in the miRNA Pathway. Int J Mol Sci 17, doi: 10.3390/ijms17010031 (2015). PMC4730277 26712751

[b26] Kedde M. & Agami R. Interplay between microRNAs and RNA-binding proteins determines developmental processes. Cell Cycle 7, 899–903 (2008). 18414021 10.4161/cc.7.7.5644

[b27] Balkhi M. Y. . miR-29 acts as a decoy in sarcomas to protect the tumor suppressor A20 mRNA from degradation by HuR. Sci Signal 6, ra63, doi: 10.1126/scisignal.2004177 (2013). 23901138 PMC3885907

[b28] Venables J. P. . RBMY, a probable human spermatogenesis factor, and other hnRNP G proteins interact with Tra2beta and affect splicing. Hum Mol Genet 9, 685–694 (2000). 10749975 10.1093/hmg/9.5.685

[b29] de Jong O. G. . Cellular stress conditions are reflected in the protein and RNA content of endothelial cell-derived exosomes. Journal of extracellular vesicles 1, doi: 10.3402/jev.v1i0.18396 (2012). PMC3760650 24009886

[b30] Liang B. . Characterization and proteomic analysis of ovarian cancer-derived exosomes. Journal of proteomics 80, 171–182, doi: 10.1016/j.jprot.2012.12.029 (2013). 23333927

[b31] Adamik B. . An association between RBMX, a heterogeneous nuclear ribonucleoprotein, and ARTS-1 regulates extracellular TNFR1 release. Biochem Biophys Res Commun 371, 505–509, doi: 10.1016/j.bbrc.2008.04.103 (2008). 18445477 PMC2442711

[b32] Hwang H. W., Wentzel E. A. & Mendell J. T. Cell-cell contact globally activates microRNA biogenesis. Proc Natl Acad Sci USA 106, 7016–7021, doi: 10.1073/pnas.0811523106 (2009). 19359480 PMC2678439

[b33] Liu X. H. . Lnc RNA HOTAIR functions as a competing endogenous RNA to regulate HER2 expression by sponging miR-331-3p in gastric cancer. Mol Cancer 13, 92, doi: 10.1186/1476-4598-13-92 (2014). 24775712 PMC4021402

[b34] Song X. . Analysing the relationship between lncRNA and protein-coding gene and the role of lncRNA as ceRNA in pulmonary fibrosis. J Cell Mol Med 18, 991–1003, doi: 10.1111/jcmm.12243 (2014). 24702795 PMC4508140

[b35] Théry C., Amigorena S., Raposo G. & Clayton A. Isolation and characterization of exosomes from cell culture supernatants and biological fluids. *Current protocols in cell biology/editorial board, Juan S. Bonifacino …* [.] Chapter 3 (2006). 10.1002/0471143030.cb0322s30 18228490

[b36] Webber J., Steadman R., Mason M. D., Tabi Z. & Clayton A. Cancer exosomes trigger fibroblast to myofibroblast differentiation. Cancer Res 70, 9621–9630, doi: 10.1158/0008-5472.CAN-10-1722 (2010). 21098712

[b37] Bailey T. L. . MEME SUITE: tools for motif discovery and searching. Nucleic Acids Res 37, W202–208, doi: 10.1093/nar/gkp335 (2009). 19458158 PMC2703892

[b38] Thomas-Chollier M. . RSAT peak-motifs: motif analysis in full-size ChIP-seq datasets. Nucleic Acids Res 40, e31, doi: 10.1093/nar/gkr1104 (2012). 22156162 PMC3287167

[b39] Kozomara A. & Griffiths-Jones S. miRBase: annotating high confidence microRNAs using deep sequencing data. Nucleic Acids Res 42, D68–73, doi: 10.1093/nar/gkt1181 (2014). 24275495 PMC3965103

[b40] Bailey T. L. DREME: motif discovery in transcription factor ChIP-seq data. Bioinformatics 27, 1653–1659, doi: 10.1093/bioinformatics/btr261 (2011). 21543442 PMC3106199

[b41] R W. Inferences based on a skipped correlation coefficient. Journal of Applied Statistics 31, 131–143 (2004).

[b42] Crooks G. E., Hon G., Chandonia J. M. & Brenner S. E. WebLogo: a sequence logo generator. Genome Res 14, 1188–1190, doi: 10.1101/gr.849004 (2004). 15173120 PMC419797

[b43] Frith M. C., Saunders N. F., Kobe B. & Bailey T. L. Discovering sequence motifs with arbitrary insertions and deletions. PLoS Comput Biol 4, e1000071, doi: 10.1371/journal.pcbi.1000071 (2008). 18437229 PMC2323616

[b44] Cheadle C., Vawter M. P., Freed W. J. & Becker K. G. Analysis of microarray data using Z score transformation. J Mol Diagn 5, 73–81, doi: 10.1016/S1525-1578(10)60455-2 (2003). 12707371 PMC1907322

