# Supplementary material for: Long non-coding RNAs harboring miRNA seed regions are enriched in prostate cancer exosomes
Source: Sci Rep. 2016 Apr 22;6:24922. doi: 10.1038/srep24922 (PMC4840345; doi:10.1038/srep24922)
Supplement: Supplementary Tables [file srep24922-s1.pdf]

Title: Long non-coding RNAs harboring miRNA seed regions are enriched in prostate cancer exosomes

Authors: Alireza Ahadi, Samuel Brennan, Paul J. Kennedy, Gyorgy Hutvagner and Nham Tran

Supplementary table S1. Overrepresentation of eight miRNA seed region motifs in highly enriched exosomal lncRNAs from the PC3 cell line. Fold change of the miRNAs is shown in the last column.

Motif	miRNA	Fold-change
AAGGAG	hsa-miR-130b	8.55
GCUGGG	miR-221-5p	12.44
UUCUUU	hsa-miR-185	8.00
CUCCUG	hsa-miR-30c	8.37
	hsa-miR-30b	7.90
CAGCCU	hsa-miR-762	9.15
UUUUUG	hsa-miR-151-5p	11.01
	hsa-miR-30c	8.37
	hsa-miR-30b	7.90
CUGGGA	hsa-miR-30d	8.67
GGCUGG	hsa-miR-149*	10.12

Supplementary table S2. List of 6-mers which are highly enriched in commonly upregulated exosomal transcripts compare to the cell.

Motif	Count difference in two sets	Number of miRNA with a perfect seed match to motif
CCUCCC	121	10
GCCUCC	116	13
UUUUCU	109	13
UGUGUG	109	38
AGCCUC	102	12
UUUCUU	102	76
UUCUUU	100	81
CUCCUG	97	25
UUUUUG	94	76
UUUUUA	92	48
CUUUUU	89	59
GCUGGG	88	14
CUCCCA	87	15
UCUUUU	86	63
GUGUGU	86	24
UGUUUU	82	127
AAUUUU	80	12
CUGGGA	79	11
CUGCCU	78	23
CAGCCU	74	11
UCUGCC	73	19
UCCCAA	73	8
UUUUGU	72	53
CCUCAG	72	11
CCUCCU	70	16
CUCAGC	69	7
CCUGGG	69	16
CCCAAG	68	2
GUGUGA	67	16
GGCUGG	67	21
CUGGAG	66	16
GCCCAG	66	9
CCUGCC	65	11
AGUGUG	65	8
GAGCCU	65	8
AGGCUG	65	10
UUUGUU	64	45
AGCUGG	63	9
UUUUAA	63	15
UUUUAU	62	29

Supplementary table S5A: Transcripts enriched in the exosomes with extreme fold change values in at least one cell line.

Transcript accession ID	VCaP	LNCaP	DU145	PC3	Long ncRNA type
ENST00000429882	5.74	< 2.0	235.21	< 2.0	intronic antisense
ENST00000501561	66.42	5.20	5.40	< 2.0	intronic antisense
ENST00000514124	65.09	< 2.0	19.06	8.46	intergenic
ENST00000501280	61.98	21.43	3.17	14.74	bidirectional
ENST00000499638	60.02	4.74	< 2.0	< 2.0	intronic antisense
ENST00000397737	57.85	< 2.0	8.65	< 2.0	intergenic
Z41842	57.35	< 2.0	6.97	2.22	intergenic
CR624806	54.39	7.17	< 2.0	< 2.0	natural antisense
NR_033396	54.30	3.31	3.57	< 2.0	exon sense-overlapping
AK097298	52.04	< 2.0	2.32	< 2.0	intergenic
ENST00000418607	50.41	12.53	< 2.0	2.83	intergenic
uc001qgn.1	50.01	5.44	2.87	3.02	intronic antisense
ENST00000424052	49.76	16.49	< 2.0	< 2.0	intergenic
ENST00000512947	49.08	2.10	2.70	< 2.0	intergenic
ENST00000453968	48.46	2.45	2.30	2.32	intergenic
ENST00000447412	46.75	< 2.0	2.29	< 2.0	intergenic
CB219568	46.53	< 2.0	6.83	< 2.0	intergenic
G36642	45.96	3.04	3.05	2.18	intronic antisense
NR_029401	45.95	< 2.0	< 2.0	< 2.0	intergenic
AL133652	45.35	< 2.0	2.19	< 2.0	intergenic
AW452601	45.14	40.23	< 2.0	< 2.0	intergenic
CA433478	44.82	< 2.0	2.78	< 2.0	intergenic
BX647519	44.81	< 2.0	2.48	< 2.0	intergenic
HIT000070262	44.17	2.74	2.57	2.14	natural antisense
uc003slj.1	43.99	2.51	2.91	< 2.0	intronic antisense
ENST00000509343	43.57	8.82	< 2.0	< 2.0	intronic antisense
uc001lnz.2	43.18	< 2.0	3.35	2.04	intergenic
AK055050	43.06	4.12	< 2.0	< 2.0	intergenic
ENST00000445662	42.81	3.41	2.09	< 2.0	intergenic
ENST00000507842	42.43	< 2.0	< 2.0	< 2.0	intronic antisense
ENST00000414425	42.37	< 2.0	< 2.0	< 2.0	intergenic
uc002aug.2	41.74	< 2.0	4.86	< 2.0	intronic antisense
ENST00000424521	40.87	2.52	2.79	< 2.0	intergenic
BC032036	40.65	< 2.0	< 2.0	< 2.0	natural antisense
AK056249	40.20	< 2.0	< 2.0	< 2.0	intronic antisense
NR_024362	40.16	3.03	< 2.0	< 2.0	intergenic
NR_003034	39.67	< 2.0	8.59	< 2.0	intergenic
ENST00000448674	39.24	4.32	< 2.0	< 2.0	intergenic
AK055500	39.12	3.47	3.16	6.14	intergenic
ENST00000507684	38.44	< 2.0	3.06	< 2.0	natural antisense
chr4:15911552-15921927-	37.91	3.05	2.60	< 2.0	intergenic
chr2:151609179-151621879+	36.57	2.49	2.60	< 2.0	intergenic
NR_023388	< 2.0	< 2.0	35.79	< 2.0	intron sense-overlapping
nc-HOXC12-103-	34.81	2.42	5.17	< 2.0	intergenic
AK124090	34.51	2.00	3.42	< 2.0	intergenic
NR_029455	33.90	2.26	3.16	< 2.0	intergenic
ENST00000414296	33.85	< 2.0	2.20	< 2.0	intergenic
uc003nbk.2	33.09	< 2.0	2.87	< 2.0	intronic antisense
uc002nqy.1	32.38	< 2.0	3.89	< 2.0	intergenic
ENST00000503372	32.23	< 2.0	< 2.0	< 2.0	intergenic
AK074614	32.12	2.18	2.49	< 2.0	natural antisense
uc003kmo.1	32.11	< 2.0	5.71	< 2.0	exon sense-overlapping
BC047297	32.01	2.61	< 2.0	< 2.0	intergenic
ENST00000424045	32.00	< 2.0	3.89	< 2.0	intergenic
ENST00000503483	31.91	2.11	< 2.0	< 2.0	bidirectional
ENST00000440744	31.67	2.09	2.07	< 2.0	intergenic
ENST00000513378	31.64	< 2.0	3.77	< 2.0	intronic antisense
ENST00000461943	31.62	2.15	2.36	< 2.0	natural antisense
NR_002728	31.21	< 2.0	7.82	< 2.0	natural antisense
uc001mwl.2	30.69	< 2.0	7.91	< 2.0	intronic antisense
AL117549	30.63	2.32	2.30	< 2.0	natural antisense
AY545553	30.51	6.23	< 2.0	3.17	natural antisense
ENST00000439157	30.46	< 2.0	< 2.0	< 2.0	intergenic
uc010mkn.1	30.25	8.58	< 2.0	5.58	intergenic
AF087972	29.89	< 2.0	7.32	< 2.0	intergenic
ENST00000502182	29.50	< 2.0	< 2.0	< 2.0	natural antisense
chr11:102425615-102440915+	29.45	< 2.0	< 2.0	< 2.0	intergenic
ENST00000430667	29.41	5.34	< 2.0	< 2.0	intergenic

ENST00000410685	29.35	2.55	2.00	< 2.0	intergenic
ENST00000446213	28.84	< 2.0	4.86	< 2.0	intergenic
uc010bys.1	28.74	11.04	2.55	14.63	intergenic
AX765693	28.62	< 2.0	2.37	< 2.0	natural antisense
AK022274	28.56	< 2.0	3.30	< 2.0	intergenic
ENST00000435658	28.56	2.65	2.23	< 2.0	intergenic
HIT000324966	28.46	< 2.0	3.65	2.24	natural antisense
HIT000221810_04	28.31	< 2.0	7.57	< 2.0	intergenic
ENST00000412381	28.00	2.36	2.70	< 2.0	intergenic
NR_026682	27.98	2.35	< 2.0	< 2.0	bidirectional
AK123278	27.88	6.05	< 2.0	< 2.0	intronic antisense
ENST00000404319	27.85	< 2.0	< 2.0	< 2.0	intronic antisense
ENST00000485404	< 2.0	27.85	< 2.0	< 2.0	intergenic
NR_015379	< 2.0	< 2.0	27.62	< 2.0	intergenic
nc-HOXC10-120-	4.04	27.51	< 2.0	< 2.0	intergenic
uc.345-	27.45	< 2.0	2.17	< 2.0	natural antisense
uc004cfy.2	27.24	3.66	< 2.0	< 2.0	natural antisense
AK021990	< 2.0	26.98	< 2.0	2.63	intronic antisense
chrX:46050831-46067706-	26.70	< 2.0	< 2.0	< 2.0	intergenic
BC044852	26.37	2.66	< 2.0	< 2.0	intronic antisense
NR_024168	26.32	< 2.0	5.55	< 2.0	exon sense-overlapping
NR_029429	26.30	< 2.0	2.38	2.76	intergenic
BE006786	26.16	5.59	< 2.0	< 2.0	intergenic
NR_024554	26.11	< 2.0	< 2.0	< 2.0	exon sense-overlapping
ENST00000458366	25.99	7.26	< 2.0	4.78	intergenic
ENST00000425911	25.81	5.53	< 2.0	2.80	intergenic
ENST00000440160	25.61	2.17	2.61	< 2.0	intergenic
CR613822	< 2.0	< 2.0	25.42	< 2.0	bidirectional
ENST00000449830	25.41	3.21	< 2.0	< 2.0	bidirectional
AI621345	25.24	2.09	4.35	< 2.0	intergenic
AK125430	25.19	< 2.0	5.94	< 2.0	intergenic
chr12:114662611-114678879+	4.31	< 2.0	23.97	< 2.0	intergenic
G30695	2.39	23.31	< 2.0	2.23	intronic antisense
ENST00000427591	6.81	23.06	< 2.0	3.16	intergenic
EB388187	11.57	< 2.0	< 2.0	18.28	intergenic
AB073602	5.31	< 2.0	< 2.0	17.70	intergenic
ENST00000485873	< 2.0	< 2.0	< 2.0	15.60	intergenic
ENST00000394732	< 2.0	< 2.0	< 2.0	14.95	intergenic
BG218391	2.21	< 2.0	2.84	13.75	intergenic
ENST00000497538	< 2.0	< 2.0	< 2.0	13.67	intergenic
ENST00000434398	6.91	4.79	< 2.0	13.46	intergenic
uc003vcq.1	< 2.0	< 2.0	2.56	12.16	natural antisense
ENST00000492981	5.69	4.63	< 2.0	12.05	natural antisense
BC059172	12.36	8.92	< 2.0	12.03	intronic antisense
ENST00000512341	3.65	< 2.0	4.59	11.45	intergenic
ENST00000401037	< 2.0	< 2.0	2.43	11.15	bidirectional
ENST00000477387	< 2.0	< 2.0	< 2.0	10.89	intronic antisense
AK021773	10.72	< 2.0	5.90	10.51	intronic antisense
ENST00000414126	< 2.0	< 2.0	< 2.0	10.48	intergenic
ENST00000433950	2.81	< 2.0	2.61	10.46	intergenic
NR_027169	2.15	< 2.0	< 2.0	10.23	natural antisense
chr1:112541627-112559477-	5.36	2.09	< 2.0	10.05	intergenic
nc-HOXC11-113-	< 2.0	3.38	< 2.0	9.57	intergenic
ENST00000458080	< 2.0	< 2.0	2.16	9.55	bidirectional
ENST00000399586	< 2.0	< 2.0	< 2.0	9.44	intergenic
NR_027667	< 2.0	< 2.0	< 2.0	9.36	natural antisense
ENST00000441206	< 2.0	< 2.0	< 2.0	9.11	intergenic
NR_027179	< 2.0	< 2.0	2.21	8.76	intergenic
ENST00000511640	2.44	< 2.0	< 2.0	8.50	intergenic
ENST00000499690	15.39	16.23	2.01	8.46	intronic antisense
uc003vcp.1	< 2.0	< 2.0	< 2.0	8.43	intronic antisense
CD514359	5.70	6.09	< 2.0	8.41	intergenic
uc.203+	< 2.0	< 2.0	< 2.0	8.36	exon sense-overlapping
BC034791	14.93	< 2.0	2.05	8.32	intergenic
uc011mlh.1	< 2.0	< 2.0	< 2.0	8.31	intergenic
ENST00000509693	2.64	< 2.0	< 2.0	8.10	bidirectional
NR_024385	6.89	5.10	< 2.0	7.97	intergenic
AK055160	< 2.0	< 2.0	< 2.0	7.68	exon sense-overlapping
ENST00000452932	11.77	9.53	2.34	7.67	intergenic
uc002xjn.1	4.76	2.48	< 2.0	7.63	natural antisense
NR_027167	< 2.0	< 2.0	< 2.0	7.37	natural antisense

uc003vcr.1	< 2.0	< 2.0	3.24	7.35	natural antisense
ENST00000402379	2.92	< 2.0	< 2.0	7.33	intergenic

Supplementary table S5B: Transcripts enriched in the cells with extreme fold change values in at least one cell line.

Transcript accession ID	VCaP	LNCaP	DU145	PC3	Long ncRNA type
BC024102	96.30	< 2.0	2.16	3.11	intergenic
ENST00000429327	86.21	2.37	< 2.0	2.44	intergenic
ENST00000415303	81.85	< 2.0	2.09	< 2.0	intergenic
ENST00000514397	74.62	3.20	< 2.0	6.47	intergenic
ENST00000441633	74.45	< 2.0	2.01	2.72	intergenic
ENST00000435146	70.90	< 2.0	2.11	< 2.0	intergenic
uc001aaz.2	69.93	< 2.0	2.10	3.49	intergenic
ENST00000511037	69.83	< 2.0	< 2.0	2.99	intronic antisense
uc004cox.3	63.89	< 2.0	2.76	15.41	intergenic
ENST00000446263	61.80	< 2.0	2.37	2.04	intergenic
ENST00000440805	60.19	< 2.0	< 2.0	< 2.0	intergenic
uc004coq.3	57.54	3.05	< 2.0	2.82	intergenic
ENST00000501056	55.33	< 2.0	< 2.0	3.19	intronic antisense
ENST00000424002	54.44	< 2.0	2.05	< 2.0	intergenic
ENST00000435196	54.44	5.08	< 2.0	6.59	intergenic
ENST00000426710	53.07	< 2.0	< 2.0	6.59	intergenic
AK022164	52.82	< 2.0	4.55	12.47	intron sense-overlapping
ENST00000416914	48.80	< 2.0	< 2.0	< 2.0	intergenic
AK098134	48.67	3.59	2.92	14.14	intergenic
AK129699	48.17	9.63	< 2.0	6.52	intergenic
ENST00000450770	47.77	4.36	< 2.0	13.92	intergenic
AK024147	45.88	2.54	< 2.0	12.93	intron sense-overlapping
HIT000273064	44.70	< 2.0	< 2.0	< 2.0	intergenic
uc002gzc.1	41.94	< 2.0	< 2.0	3.85	intergenic
AK096384	39.42	< 2.0	< 2.0	5.26	intergenic
NR_002569	38.71	2.10	< 2.0	3.88	intron sense-overlapping
ENST00000514978	38.48	< 2.0	< 2.0	2.03	intergenic
AK093407	38.40	< 2.0	2.96	4.52	intergenic
uc001aba.1	37.41	< 2.0	2.67	3.22	intergenic
NR_003013	37.36	< 2.0	< 2.0	< 2.0	intron sense-overlapping
NR_003004	36.74	< 2.0	< 2.0	< 2.0	intron sense-overlapping
NR_004388	36.56	< 2.0	< 2.0	< 2.0	intron sense-overlapping
ENST00000509243	36.17	< 2.0	2.25	2.54	intronic antisense
ENST00000512617	34.70	< 2.0	< 2.0	7.12	intergenic
ENST00000509155	33.35	< 2.0	2.30	2.53	intergenic
AK024108	33.13	< 2.0	2.92	15.17	intron sense-overlapping
ENST00000435623	33.03	2.02	< 2.0	4.32	intergenic
ENST00000484956	32.74	< 2.0	< 2.0	2.23	intergenic
NR_027330	30.41	< 2.0	< 2.0	21.14	intron sense-overlapping
ENST00000498999	30.07	< 2.0	< 2.0	< 2.0	intronic antisense
AK000561	28.57	< 2.0	< 2.0	2.63	intergenic
NR_003012	25.86	< 2.0	< 2.0	< 2.0	intron sense-overlapping
NR_023388	16.57	8.21	< 2.0	25.74	intron sense-overlapping
AK021558	24.75	4.51	< 2.0	6.56	bidirectional
NR_024456	24.19	3.84	< 2.0	4.37	intergenic
uc004exm.2	7.22	2.18	8.78	22.27	intergenic
uc.203+	< 2.0	21.42	< 2.0	< 2.0	exon sense-overlapping
NR_027330	30.41	< 2.0	< 2.0	21.14	intron sense-overlapping
U79293	6.93	6.67	< 2.0	20.41	intron sense-overlapping
uc003jka.1	10.24	< 2.0	< 2.0	19.39	intergenic
ENST00000441241	22.57	< 2.0	< 2.0	18.60	intergenic
CR590180	14.12	< 2.0	4.73	18.56	bidirectional
ENST00000412709	5.10	18.21	< 2.0	< 2.0	intronic antisense
CR605514	4.57	< 2.0	< 2.0	17.65	intergenic
AK125665	7.63	< 2.0	< 2.0	16.98	intronic antisense
NR_027487	8.51	5.87	< 2.0	16.87	intergenic
HIT000191751	< 2.0	2.08	< 2.0	16.26	intergenic
NR_024618	3.34	< 2.0	< 2.0	16.24	intergenic
AK130332	< 2.0	16.01	< 2.0	< 2.0	intergenic
NR_028324	2.84	< 2.0	< 2.0	15.98	intergenic
uc004cox.3	63.90	< 2.0	2.76	15.42	intergenic
NR_027439	19.57	< 2.0	< 2.0	15.26	intergenic
uc001dbm.2	7.19	< 2.0	3.52	14.49	intergenic
NR_027345	8.05	6.36	< 2.0	14.49	intergenic
AK090477	22.41	< 2.0	< 2.0	13.93	intergenic
uc003srb.1	6.42	< 2.0	< 2.0	13.36	exon sense-overlapping
BC032761	3.90	< 2.0	< 2.0	13.27	intergenic
ENST00000415467	< 2.0	13.19	< 2.0	< 2.0	bidirectional

ENST00000450885	3.07	< 2.0	< 2.0	13.12	intergenic
ENST00000439050	< 2.0	13.02	< 2.0	< 2.0	intergenic
BC016339	19.92	3.10	< 2.0	12.75	natural antisense
NR_015379	2.39	12.46	< 2.0	< 2.0	intergenic
BC013423	2.33	< 2.0	2.04	12.45	intergenic
BC005232	10.64	< 2.0	< 2.0	12.30	exon sense-overlapping
AL359062	< 2.0	11.98	< 2.0	12.20	intergenic
NR_027447	3.85	4.15	< 2.0	12.08	bidirectional
ENST00000456305	3.14	11.94	< 2.0	< 2.0	intergenic
CR597075	15.22	< 2.0	2.30	11.88	exon sense-overlapping
AK096223	5.81	11.75	< 2.0	6.52	natural antisense
AK057652	18.71	3.06	< 2.0	11.66	intergenic
ENST00000398262	< 2.0	11.66	< 2.0	< 2.0	intergenic
ENST00000515248	6.36	< 2.0	< 2.0	11.56	intergenic
AK090904	3.02	< 2.0	< 2.0	11.55	intergenic
ENST00000413071	< 2.0	11.38	< 2.0	< 2.0	bidirectional
uc003ghh.3	< 2.0	3.63	< 2.0	11.29	intergenic
uc003szc.1	3.48	2.18	< 2.0	11.27	bidirectional
AK055014	4.60	< 2.0	< 2.0	11.18	natural antisense
ENST00000421806	< 2.0	< 2.0	< 2.0	11.16	intergenic
BC041955	< 2.0	11.14	< 2.0	3.79	intergenic
uc.186-	4.44	< 2.0	11.10	< 2.0	exon sense-overlapping
CR601061	5.67	2.26	< 2.0	10.82	intergenic
CR603951	< 2.0	3.44	< 2.0	10.49	intergenic
uc002psm.1	3.41	< 2.0	< 2.0	10.49	intergenic
ENST00000322209	< 2.0	10.34	< 2.0	< 2.0	intergenic
DQ372724	2.40	< 2.0	< 2.0	10.28	intergenic
nc-HOXA13-99+	3.46	< 2.0	< 2.0	10.24	intergenic
ENST00000397737	< 2.0	10.23	< 2.0	< 2.0	intergenic
AL365520	< 2.0	< 2.0	< 2.0	10.21	intergenic
ENST00000374922	6.65	10.15	< 2.0	< 2.0	intergenic
ENST00000413496	12.84	< 2.0	< 2.0	10.00	natural antisense
uc002ttc.1	7.46	3.04	< 2.0	9.97	natural antisense
NR_026710	7.22	< 2.0	< 2.0	9.92	natural antisense
ENST00000501658	5.29	9.85	< 2.0	< 2.0	natural antisense
BX649150	15.57	< 2.0	4.79	9.80	intergenic
AK094163	< 2.0	< 2.0	5.67	9.79	intergenic
AK023096	< 2.0	< 2.0	< 2.0	9.75	exon sense-overlapping
uc002bow.2	< 2.0	< 2.0	< 2.0	9.74	intronic antisense
ENST00000504184	8.19	5.30	< 2.0	9.73	intergenic
uc.63-	18.62	3.47	3.34	9.65	intron sense-overlapping
NR_027064	3.65	9.54	< 2.0	3.07	intergenic
ENST00000426314	3.31	< 2.0	< 2.0	9.53	intergenic
BC008846	10.18	< 2.0	< 2.0	9.48	intergenic
uc010fsr.1	3.40	3.99	< 2.0	9.45	natural antisense
AK124497	5.13	9.40	< 2.0	3.68	natural antisense
NR_027715	11.12	< 2.0	5.19	9.37	intron sense-overlapping
uc003fax.1	< 2.0	9.35	< 2.0	< 2.0	intergenic
uc001zgs.1	2.30	9.30	< 2.0	< 2.0	intergenic
NR_027046	10.79	5.63	< 2.0	9.24	intergenic
uc002qim.1	4.35	2.10	< 2.0	9.19	intergenic
ENST00000483609	5.80	< 2.0	< 2.0	9.16	intergenic
nc-HOXC10-120-	< 2.0	< 2.0	2.07	9.04	intergenic
ENST00000425209	2.52	8.98	< 2.0	< 2.0	intergenic
NR_023387	3.82	8.96	< 2.0	3.03	intergenic
ENST00000440184	3.27	8.89	< 2.0	< 2.0	intergenic
ENST00000411597	10.77	8.86	< 2.0	< 2.0	intergenic
ENST00000494640	< 2.0	8.84	2.53	< 2.0	intergenic
ENST00000512165	< 2.0	8.83	< 2.0	< 2.0	intergenic
uc004exm.2	7.22	2.18	8.78	22.27	intergenic
ENST00000412427	3.34	< 2.0	8.61	< 2.0	intergenic
NR_002217	< 2.0	8.55	< 2.0	< 2.0	intergenic
ENST00000453697	11.97	< 2.0	8.50	< 2.0	intergenic
ENST00000423943	< 2.0	8.44	< 2.0	< 2.0	intergenic
CR601404	5.22	8.43	< 2.0	< 2.0	natural antisense
ENST00000511618	3.36	8.33	< 2.0	< 2.0	intergenic
BC033643	5.20	8.31	< 2.0	3.61	natural antisense
ENST00000501107	4.81	8.18	< 2.0	5.45	intergenic
AK026820	2.57	8.17	< 2.0	5.76	intron sense-overlapping
ENST00000423298	< 2.0	< 2.0	8.17	< 2.0	intergenic
uc002jsc.1	< 2.0	8.05	< 2.0	< 2.0	intergenic

AL137346	< 2.0	< 2.0	7.99	< 2.0	intergenic
U85044	6.74	7.91	< 2.0	< 2.0	natural antisense
ENST00000451357	< 2.0	7.86	< 2.0	< 2.0	intergenic
AK125321	3.16	< 2.0	7.59	2.49	natural antisense
AK130741	< 2.0	< 2.0	7.28	5.43	intergenic
BC017989	2.69	< 2.0	6.64	4.24	intronic antisense
ENST00000460407	2.39	< 2.0	6.42	< 2.0	intergenic
ENST00000478252	3.29	< 2.0	6.14	< 2.0	intronic antisense
ENST00000419271	< 2.0	< 2.0	6.11	< 2.0	intergenic
AK125416	< 2.0	< 2.0	5.84	< 2.0	intronic antisense
ENST00000511640	< 2.0	4.06	5.75	< 2.0	intergenic
ENST00000416072	2.17	< 2.0	5.67	2.09	natural antisense
